# The relationship between gastric microbiome features and responses to neoadjuvant chemotherapy in gastric cancer

**DOI:** 10.3389/fmicb.2024.1357261

**Published:** 2024-04-17

**Authors:** Peng Zhang, Jianfei Xu, Yanbing Zhou

**Affiliations:** ^1^Department of Gastrointestinal Surgery, Affiliated Hospital of Qingdao University, Qingdao, China; ^2^Department of Emergency Surgery, Affiliated Hospital of Qingdao University, Qingdao, China

**Keywords:** Shandong provincial natural science foundation, China (no. ZR2021MH001) gastric cancer, neoadjuvant chemotheraphy, gastric microbiome, 16S rRNA sequencing, gastric cancer

## Abstract

**Background:**

Emerging evidence demonstrates that the gastrointestinal microbiome has the potential to be a biomarker in neoadjuvant chemoradiotherapy for colorectal cancer (CRC). Yet studies on the impact of the gastric microbiome (GM) on the response to neoadjuvant chemotherapy (NACT) are still scarce.

**Methods:**

Forty-eight patients with gastric cancer participated in this retrospective study, and 16S rRNA sequencing was performed to evaluate formalin-fixed and paraffin-embedded (FFPE) tissue biospecimens and fresh-frozen tissues.

**Results:**

In this study, 16 bacterial taxa at different levels, including *Bacillus*, *Anaerococcus*, and *Chloroflexi*, were identified to be enriched before NACT in response (R) patients in group FFPE. In contrast, 6 bacterial taxa, such as *Haemophilus*, *Veillonellaceae* (*Veillonella*), etc. were enriched after NACT, in which we reported for the first time that the phylum *Chloroflexi* was enriched before NACT in R patients. Thirty-one bacterial taxa of *Coriobacteriaceae*, *Ruminococcaceae*, *Veillonellaceae*, and *Lachnospiraceae* were identified in group mucosa as being enriched in R patients. In comparison, 4 bacterial taxa dominated by the phylum *Proteobacteria* were enriched in NR patients. Notably, the family *Veillonellaceae* was found in both tissue samples, and the metabolic pathways, including the citrate cycle (TCA cycle) and various amino acids, including alanine, were found to be potentially predictive in both sample species.

**Conclusion:**

There are differences in the features of the GM for different NACT response results. The causal relationship deserves to be confirmed by further investigations.

## Introduction

Gastric cancer (GC) is a global disease, with more than 100,000 new cases estimated each year (Bray et al., 2018). It is the fifth most common malignant tumor worldwide and the third leading cause of cancer-related deaths, with 784,000 deaths worldwide in 2018 (Bray et al., 2018). In China, GC is the second most common cancer and the second leading cause of cancer-related deaths (Cao et al., 2021). GC has a higher mortality and 5-year prevalence rate in China compared with most developed countries, and more worryingly, its incidence has been on the rise in the younger population (Cao et al., 2021).

Surgery, including surgical or endoscopic resection, is the mainstay of its treatment (Smyth et al., 2020). Among these options, radical gastric cancer surgery with D2 lymph node dissection has become the standard procedure for advanced gastric cancer (AGC) (Japanese Gastric Cancer Association, 2021). However, its mortality rate remains high because GC is mostly advanced at the time of diagnosis (Bray et al., 2018; Cao et al., 2021). Notably, neoadjuvant chemotherapy (NACT) increases AGC’s chances of resection cure. Additionally, the advantages of NACT are gradually recognized, such as lowering tumor stage, eliminating occult micrometastases, and increasing the chances of radical resection (Cunningham et al., 2006; Smyth et al., 2020). The Chinese Society of Clinical Oncology (CSCO) suggests that NACT may be considered for patients with advanced resectable GC (stage cIII or greater) (Wang et al., 2021). Furthermore, the European Society for Medical Oncology (ESMO) treatment guidelines (Smyth et al., 2016) strongly recommend platinum or fluoropyrimidines in combination with perioperative (preoperative and postoperative) chemotherapy for patients with stage 1B or more resectable GC.

The effect of NACT varies from person to person. The influencing factors include cell-intrinsic mechanisms such as drug transport, autophagy, apoptosis inhibition, DNA damage repair, genomic instability, and cell-extrinsic factors such as tumor microenvironment (TME) (Zheng, 2017; Hekmatshoar et al., 2018). In recent years, the influence of commensal bacteria in the metabolism, efficacy, and toxicity of chemotherapeutic drugs has also been reported (Li et al., 2016; Yamamura et al., 2019; Jang et al., 2020; Li et al., 2021; Yi et al., 2021; Teng et al., 2023). However, the relationship between the microbiota of the stomach and NACT for GC has rarely been explored due to its unique, strongly acidic environment (Stewart et al., 2020). Infection with *Helicobacter pylori* is widely recognized as the greatest risk factor for gastric carcinogenesis (Wroblewski et al., 2010). Other than that, no particular bacteria species is associated with the progression or treatment of GC.

This study collected and analyzed FFPE tissue biospecimens and tumor mucosa samples from GC patients using 16S rRNA sequencing to investigate the relationship between gastric microbiome (GM) and neoadjuvant chemotherapy (NACT) responses in GC patients.

## Materials and methods

### Study design and project

We retrospectively collected samples from 48 GC patients from January 2017 to January 2023, categorized into groups FFPE and mucosa by differences in sample preservation methods, 17 and 31, respectively. Group FFPE samples were collected from GC patients before and after NACT, while group mucosa samples were only collected from postoperative fresh frozen tissues after NACT. The above patients met the inclusion and exclusion criteria of this study, i.e., patients with a pathologically confirmed diagnosis of gastric adenocarcinoma, without metastasis or other primary tumors, who received only NACT before surgery and successfully underwent radical gastric cancer surgery were included in this study. The presence of metastasis or other primary tumor lesions, receiving NACT without radical gastric cancer surgery, and receiving other conversion therapies, including immunotherapy, targeted therapy, and probiotics within 1 month before surgery, were excluded from this study. FFPE samples were obtained from the sample bank of the Department of Pathology of the center; GC tumor mucosa samples were obtained from the tumor sample bank of the Department of Gastrointestinal Surgery of the center, and the tumor samples were frozen and stored at −80°C immediately after 20 min of *ex vivo*. The collection of samples was done in full compliance with the Implementing Rules of the Regulations for the Management of Human Genetic Resources. According to the guidelines, different NACT regimens were given according to the individual tolerance differences of patients, usually including conventional chemotherapy regimens such as 5-F, oxaliplatin, and capecitabine. Samples were then processed and analyzed using 16S Ribosomal RNA Gene Sequencing. To assess NACT efficacy, the 8th edition of the American Joint Committee on Cancer (AJCC) staging system and the College of American Pathologists (CAP) four-point tumor regression grading system were used to grade patients with gastric cancer. Patients were classified as “response” (R) if postoperative tumor regression grade (TRG) pathology was reported as TRG 0–1, ypT0-1, and ypN0, and as “no response” (NR) if TRG2-3, ypT2-4, or ypN+ (Ryan et al., 2005; Yi et al., 2021). The study was approved by the Ethics Committee of the Affiliated Hospital of Qingdao University, and all the participants signed an informed consent form.

### 16S ribosomal RNA sequencing

Genomic DNA was extracted from fresh frozen tissues using the AllPrep DNA/RNA/miRNA Universal Kit (Qiagen, Hilden, Germany). Genomic DNA from FFPE tissues was extracted using the QIAamp DNA FFPE Tissue Kit (Qiagen). Following PCR amplification, the PCR amplification products were purified using Agencourt AMPure XP magnetic beads and dissolved in an Elution Buffer. Subsequently, the fragment ranges and concentrations of the libraries were detected using an Agilent 2,100 Bioanalyzer. Following library construction, an Agilent 2,100 Bioanalyzer was used to detect the fragment range and concentration of the library. The library was selected for sequencing on the Illumina HiSeq platform according to the insert size.

### Bioinformatics processing and analysis

Raw sequencing data were processed for quality control using the software iTools Fqtools fqcheck (v.0.25), cutadapt (v.2.6), and readfq (v1.0), and clean data was obtained and used for subsequent bioinformatics analysis (He et al., 2013). The Amplicon Sequence Variants (ASVs, 100% similar sequences) were obtained by denoising using the DADA2 (Divisive Amplicon Denoising Algorithm) method in the software QIIME2, which in turn resulted in the Feature Table (Feature). After obtaining the Feature, it was compared with the database Greengene V201305 for species annotation by RDP classifier (v2.2) software, and the confidence threshold was set to 0.6 (Wang et al., 2007). Subsequently, Alpha diversity (Simpson and Shannon) and Beta diversity analyses were performed based on the above results using the Q2-diversity plugin QIIME2. STAMP was used to identify species taxa that differed between groups with default parameters and *p* value <0.05 (Parks et al., 2014). LDA Effect Size analysis (LEfSe) was further used to compare differences in indicator species between groups (Segata et al., 2011). Subsequently, practical PICRUST2 (v2.3.0-b) software was used to predict the potential functional distribution of microbiomes (Langille et al., 2013).

### Statistical analysis

Patients’ clinical data were analyzed using SPSS version 26 software (*p* < 0.05 was considered statistically significant). The chi-square test or Fisher exact test (as appropriate) was used for qualitative variables. For quantitative variables, normality and chi-square were tested first; independent samples t-test was used for variables that conformed to normal distribution and chi-square. Otherwise, the Mann–Whitney U-test was used. Furthermore, the Wilcoxon test was used to compare Alpha and Beta diversity indices, bacterial abundance, and functional prediction analysis.

## Results

### Baseline characteristics and gastric microbiome of the study population

The clinical baseline characteristics of GC patients are shown in Table 1. Divided into group FFPE (*n* = 17) and group mucosa (*n* = 31), the former includes R (*n* = 6) and NR (*n* = 11), and the latter includes R (*n* = 12) and NR (*n* = 19). There was no statistical difference in gender, age, BMI, smoking history, tumor location, cTNM staging before NACT and chemotherapy regimens. In contrast, tumor size (group FFPE, *p* = 0.027; group mucosa, *p* = 0.001) and ypTNM staging after NACT (group FFPE, *p* = 0.006; group mucosa, *p* = 0.001) showed statistical differences in both groups. Further describing the GM changes in group FFPE patients before and after NACT, the results of 16S rRNA sequencing showed a relative diversity of GM, with insignificant changes in GM and an abundance of *Acidobacteriota*, *Deinococcota*, *Pseudomonadota*, etc. at the phylum level (Supplementary Figures 1A,B).

**Table 1:** Baseline characteristics of patients.

Baseline characteristics	Group FFPE		p*	Baseline characteristics	Group mucosa		p*
	*R* (*n* = 6)	NR (*n* = 11)			*R* (*n* = 12)	NR (*n* = 19)	
Gender			1.000	Gender			0.206
Male	4 (66.7%)	8(72.7%)		Male	7 (58.3%)	16 (84.2%)	
Female	2(33.3%)	3(27.3%)		Female	5(41.7%)	3 (15.8%)	
Age	66.00 ± 7.95	58.45 ± 10.69	0.152^#^	Age	64.00(61.50–66.00)	59.00 (51.00–65.00)	0.340^$^
BMI	25.85 (22.38–30.68)	25.26 (22.80–26.80)	0.767^#^	BMI	24.20 ± 3.08	23.65 ± 4.00	0.451^#^
Smoking history			0.644	Smoking history			0.756
Yes	3 (50.0%)	4 (36.4%)		Yes	5 (41.7%)	9 (47.4%)	
No	3 (50.0%)	7 (63.6%)		No	7 (58.3%)	10 (52.6%)	
Tumor location			0.796	Tumor location			1.000
L	4 (66.7%)	5 (45.4%)		L	8 (66.7%)	12 (63.2%)	
M	0 (0.0%)	2 (18.2%)		M	3 (25.0%)	4 (21.0%)	
U	2 (33.3%)	4 (36.4%)		U	1 (8.3%)	3 (15.8%)	
Tumor size	2.08 ± 1.11	3.82 ± 1.52	0.027^#^	Tumor size	0.75 (0.00–2.88)	3.50 (2.20–5.00)	0.001^$^
cTNM staging before NACT			1.000	cTNM staging before NACT			0.174
I	0 (0.0%)	0 (0.0%)		I	0 (0.0%)	0 (0.0%)	
II	1 (16.7%)	1 (9.1%)		II	4 (33.3%)	2 (10.5%)	
III	5 (83.3%)	10 (90.9%)		III	8 (66.7%)	17 (89.5%)	
ypTNM staging after NACT			0.006	ypTNM staging after NACT			0.001
I	4 (66.7%)	0 (0.0%)		I	7 (58.3%)	0 (0.0%)	
II	2 (33.3%)	6 (54.5%)		II	3 (25.0%)	9 (47.4%)	
III	0 (0.0%)	5 (45.5%)		III	2 (16.7%)	10 (52.6%)	
Chemotherapy regimens			0.830	Chemotherapy regimens			0.781
cisplatin+5-FU	2 (33.3%)	5 (45.4%)		cisplatin+5-FU	5 (41.7%)	9 (47.4%)	
Oxaliplatin	2 (33.3%)	4 (36.4%)		Oxaliplatin	6 (50.0%)	7 (36.8%)	
PACLITAXEL	2 (33.3%)	2 (18.2%)		Paclitaxel	1 (8.3%)	3 (15.8%)	

*Unless otherwise stated, the Pearson’s *χ*^2^ test or Fisher’s exact test was used (as appropriate); # Student’s *t*-test; $ Mann–Whitney U test. FFPE, formalin-fixed and paraffin embedded tissue biospecimen; R, response; NR, no response; BMI, body mass index; NACT, neoadjuvant chemotherapy. Data are presented in “n (%), median (range) or Mean ± SD”.

### Impact of NACT on the gastric microbiome of GC patients

To further evaluate the impact of NACT on GM in GC patients, the GM changes in group FFPE were compared. Firstly, we analyzed GM’s Alpha and Beta diversity before and after NACT and found no difference (Supplementary Figures 2A–C), indicating that NACT does not significantly change the overall diversity structure of GM. In addition, six bacterial taxa, including *Bacteroidota*, *Alphaproteobacteria*, and *Flavobacteriia*, were found to be enriched after NACT by STAMP (Supplementary Table 1). Furthermore, LefEe analysis confirmed the presence of differences in bacterial abundance in the GM before and after NACT. Among the bacterial taxa identified, 9 bacterial taxa, including *Solibacillus*, *Enterococcaceae*, and Enterococcus, were identified as enriched before NACT, and 18 bacterial taxa, including *Rhodobacterales*, *Paracoccus*, and *Bacteroidota,* were enriched following NACT (Supplementary Figures 3A,B).

### Differentials in gastric microbiome between patients with R and NR

**Figure 1:**
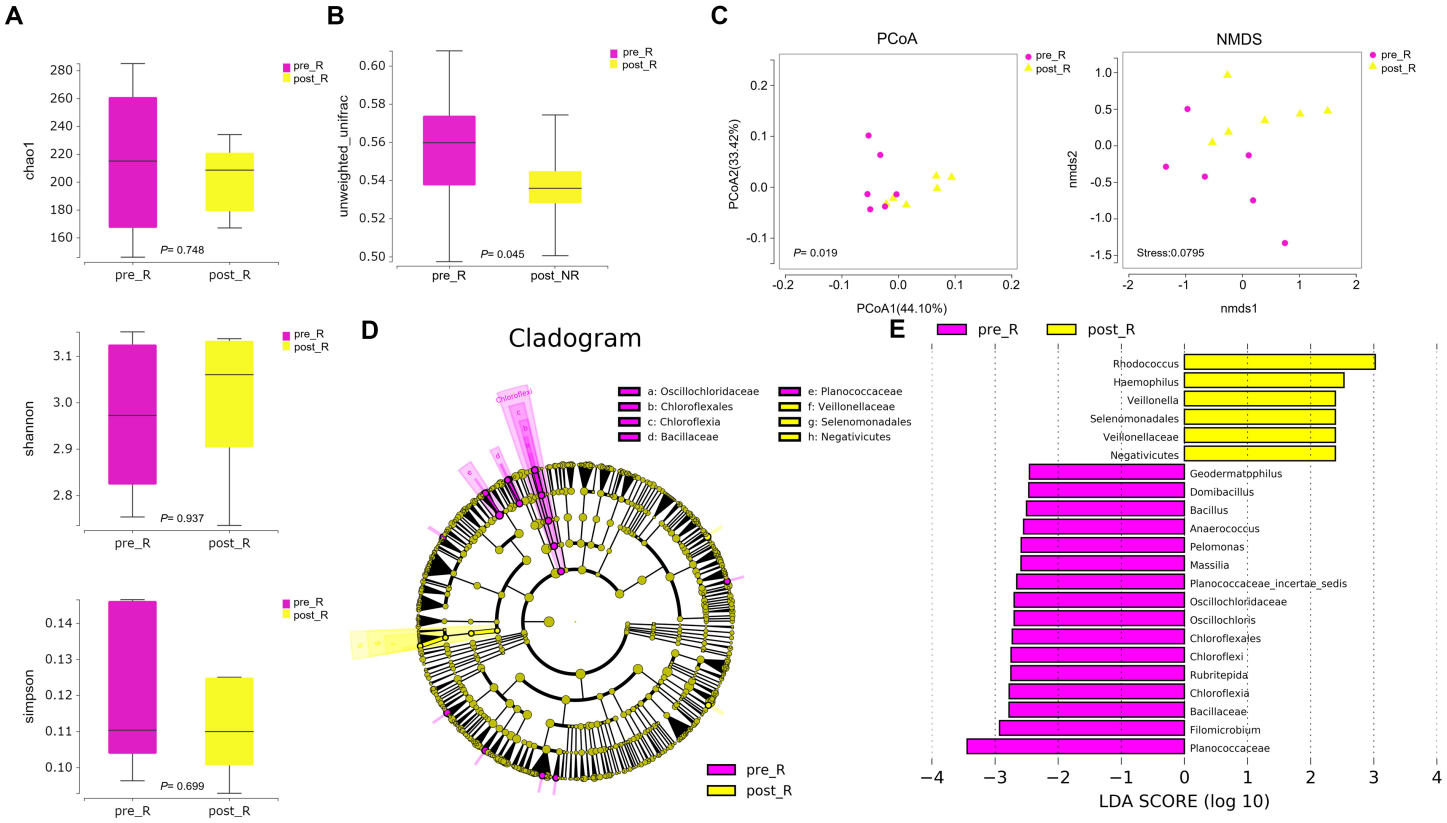
The diversity and composition of different bacterial taxa in the gastric microbiome of patients with R during NACT treatment (*n* = 6). **(A)** chao1 index, *p* = 0.748; Shannon index, *p* = 0.937; Simpson index, *p* = 0.699. The Wilcoxon Test was used and showed no Alpha diversity differences. **(B)** The gastric microbiome of R patients before and after NACT showed differences in Beta diversity (Wilcoxon Test, *p* = 0.045). **(C)** PCoA analysis showed differences (PERMANOVA Test, *p* = 0.019); NMDS analysis showed differences (PERMUTATION Test, Stress = 0.0795). **(D)** LEfSe clustered Clangrom plot. a: *Oscillochloridaceae*; b: *Chloroflexales*; c: *Chloroflexia*; d: *Bacillaceae*; e: *Planococcaceae*; f: *Veillonellaceae*; g: *Selenomonadales*; h: *Negativicutes*. **(E)** Bar chart of log_10_ LDA for bacterial differential taxa before and after NACT in R patients. Taxa listed were significant (Wilcoxon Test, *p* < 0.05; LDA scores >2). “pre_,” pre-NACT; “post_,” post-NACT; PCoA, Principal Co-ordinates Analysis; NMDS, Nonmetric Multidimensional Scaling; R, response, FFPE samples from gastric cancer patients.

GC patients’ responses to NACT differ, and the GM may be relevant (as described in the Introduction). Based on such hypotheses, we sought to determine whether there were differences in the composition and abundance of GM before and after NACT in patients with R and NR. To address this, differences in GM in group FFPE were compared. No differences in Alpha diversity were observed in either R or NR patients (Figure 1A; Supplementary Figure 4A). Differences in Beta diversity were observed in R patients (Figure 1B) but not in NR patients (Supplementary Figures 4B,C). Furthermore, PCoA and NMDS analysis verified the differences in Beta diversity in R patients (Figure 1C). Differences in specific bacterial taxa in R patients were then identified by STAMP, including 8 bacterial taxa, including *Deinococcus_Thermus*, *Bacteroidetes*, *Deinococci*, etc., enriched after NACT (Table 2). LEfSe analysis verified this difference and found that 16 bacterial taxa, including *Bacillus*, *Anaerococcus,* and C*hloroflexia,* were enriched before NACT, and 6 bacterial taxa, including *Haemophilus* and *Veillonella,* were enriched after NACT (Figures 1D,E). To further assess the possibility of certain specific bacterial taxa in R patients benefiting from NACT, GM in R and NR patients before NACT were compared for differences in microbiome composition and abundance. As expected, no differences in Alpha and Beta diversity were observed (Supplementary Figures 5A–C). However, 18 bacterial taxa, including *Planococcaceae*, *Rhizobiales*, and *Carnobacteriaceae,* were found to be enriched in R patients, and 5 bacterial taxa, including *Rhodococcus*, *Caulobacter,* and *Negativicutes,* were enriched in NR patients by STAMP and verified by LEfSe analysis (Supplementary Figures 5D,E). Moreover, we noticed that several bacterial taxa, including *Planococcaceae* (*Planococcaceae_incertae_sedis*), *Anaerococcus*, *Rubritepida*, *Geodermatophilus* (*Geodermatophiaceae*), and others, were enriched in patients with R before receiving NACT in both comparisons (Figure 1E; Supplementary Figure 5E).

**Table 2:** Comparison of gastric microbiome before and after NACT in R patients by STAMP.

Taxon	Relative Abundance of pre_R (%)	Relative Abundance of post_R (%)	*p*-values	Taxonomy level
*Deinococcus_Thermus*	27.749119	34.4645	0.02	Phylum
*Bacteroidetes*	0.796095	1.904881	0.045	Phylum
*Deinococci*	27.749119	34.4645	0.02	Class
*Sphingobacteriia*	0.298069	0.862492	0.045	Class
*Deinococcales*	26.354454	32.886653	0.031	Order
*Deinococcaceae*	26.339881	32.865551	0.031	Family
*Deinococcus*	26.339881	32.865551	0.031	Genus
*Deinococcus_murrayi*	26.235951	32.560118	0.045	Species

NACT, neoadjuvant chemotherapy; R, response; “pre_,” pre-NACT; “post_,” post-NACT.

**Figure 2:**
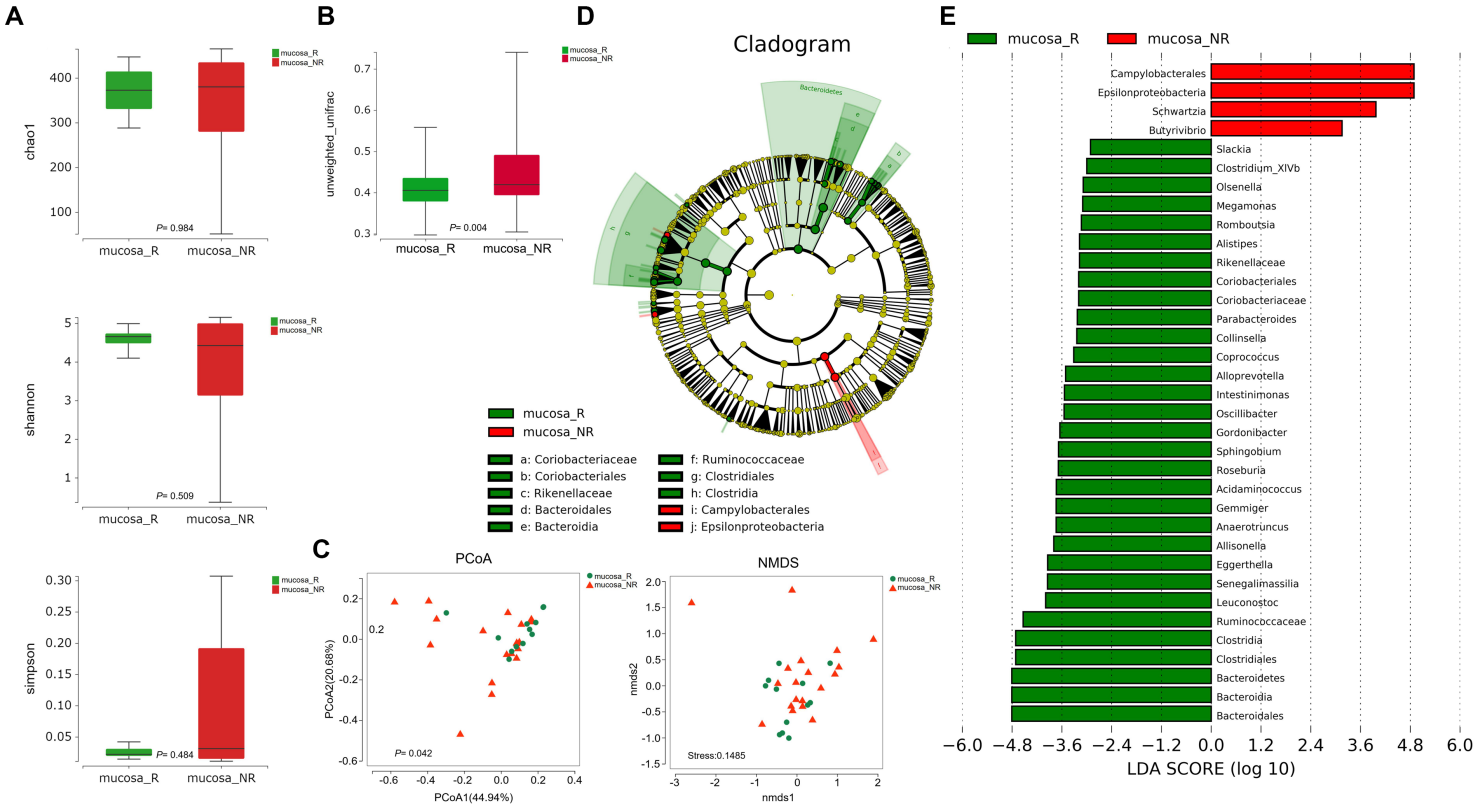
Diversity and composition of differential bacterial taxa in the gastric microbiome of patients with R (*n* = 12) and NR (*n* = 19). **(A)** Chao1 index, *p* = 0.984; Shannon index, *p* = 0.509; Simpson index, *p* = 0.484. The Wilcoxon Test was used and showed no Alpha diversity differences. **(B)** Beta diversity, *p* = 0.004 (Wilcoxon Test). **(C)** PCoA analysis showed differences (PERMANOVA Test, *p* = 0.042). B NMDS analysis showed differences (PERMUTATION Test, Stress = 0.1485). **(D)** LEfSe clustered Clangrom plot. a: *Coriobacteriaceae*; b: *Coriobacteriales*; c: *Rikenellaceae*; d: *Bacteroidales*; e: *Bacteroidia*; f: *Ruminococcaceae*; g: *Clostridiales*; h: *Clostridia*; i: *Campylobacterales*; j: *Epsilonproteobacteria*. **(E)** Bar chart of log_10_ LDA for bacterial differential taxa between R and NR patients after NACT. The taxa listed were significant (Wilcoxon Test, *p* < 0.05; LDA scores >2). PCoA, Principal Co-ordinates Analysis; NMDS, Nonmetric Multidimensional Scaling; R, response; NR, no response. FF samples from gastric cancer patients.

To better fully characterize the potential impact of GM on NACT response in GC patients, we retrospectively collected another independent cohort of gastric cancer tumor mucosa samples, namely group mucosa (Table 1). Firstly, we assessed whether there were differences in GM’s Alpha and Beta diversity between R and NR patients. The results showed no difference in Alpha diversity between the two groups (Figure 2A), while Beta diversity was significantly different (Figure 2B). Furthermore, PCoA and NMDS analysis verified the differences (Figure 2C). STAMP subsequently detected variations in specific bacterial taxa between the two groups; several bacterial taxa, including *Bacteroidetes*, *Clostridia*, *Lachnospiraceae*, *Ruminococcaceae,* etc., were enriched in patient R, whereas *Bacilli* (*Bacillus_cereus*), *Epsilonproteobacteria*, etc. were enriched in NR (Table 3). Further LEfSe analysis verified that 31 bacterial taxa, including *Coriobacteriaceae*, *Ruminococcaceae*, *Veillonellaceae*, *Lachnospiraceae*, etc., were enriched in R patients, whereas 4 bacterial taxa including *Campylobacterales* and *Epsilonproteobacteria* were enriched in NR (Figures 2D,E).

**Table 3:** Comparison of the gastric microbiome in R and NR patients after NACT by STAMP.

Taxon	Relative Abundance of mucosa_R (%)	Relative Abundance of mocusa_NR (%)	*p*-values	Taxonomy level
*Bacteroidetes*	28.19382	17.544959	0.02	Phylum
*Clostridia*	29.276092	18.397846	0.037	Class
*Bacteroidia*	28.104442	17.436246	0.02	Class
*Bacilli*	9.513941	18.570907	0.037	Class
*Epsilonproteobacteria*	5.551373	16.709311	0.037	Class
*Clostridiales*	29.276092	18.397846	0.037	Order
*Lachnospiraceae*	15.640448	10.033421	0.041	Family
*Ruminococcaceae*	10.918395	5.179571	0.03	Family
*Prevotella_copri*	16.205266	8.238723	0.033	Species
*Bacillus_cereus*	0.002455	2.679231	0.02	Species

NACT, neoadjuvant chemotherapy; R, response; NR, no response.

### Differential functional analysis of potential metabolic pathways

To identify potential functional mechanisms by which GM may influence NACT responses, KEGG enrichment analysis using PICRUST2 software predicted potential functional differences between group FFPE and group mucosa. Lipopolysaccharide (LPS) biosynthesis, Citrate cycle (TCA cycle), Glycine, serine, and threonine metabolism, etc., may be potential metabolic pathways influencing the response to NACT treatment in the former patients with R (Supplementary Figure 6, Wilcox Test, *p* < 0.05). Carbon fixation pathways in prokaryotes, D-Alanine metabolism, streptomycin biosynthesis, alanine, aspartate, and glutamate metabolism, biosynthesis of ansamycins, and citrate cycle (TCA cycle) may be potential metabolic pathways influencing the latter’s response to NACT (Supplementary Figure 7, Wilcox Test, *p* < 0.05).

## Discussion

In recent decades, increasing evidence has demonstrated that gut microbiota plays an essential role in health and disease (Stewart et al., 2020; Guo et al., 2023). Studies have shown that the gut microbiota is associated with radiotherapy for colorectal cancer^11-13^. However, the relationship between GM and NACT has rarely been reported. Moreover, many factors, such as a strongly acidic environment (Guarner and Malagelada, 2003; Stewart et al., 2020), peristaltic state (Guarner and Malagelada, 2003), *H.pylori* infection (Stewart et al., 2020), and proton pump inhibitor therapy (Amir et al., 2014), contribute to the relative instability of the GM compared to the colorectum. This, coupled with differences in study design, has led to diverse results in GM studies.

In the study, 16S rRNA sequencing was used to assess whether there were changes in the GM of patients before and after NACT. The results showed no difference in both Alpha and Beta diversity (Supplementary Figures 2A–C), which suggests that NACT may not significantly impact the overall structure of the GM in GC patients. Possible reasons include that GM escapes chemotherapeutic drugs through some evasion mechanism or that chemotherapeutic drugs do not preferentially target certain GMs. The differences in GM of R patients of group FFPE before and after NACT and between R and NR patients of group mucosa were evaluated separately. We found that at different levels, 16 bacterial taxa, including *Bacillus*, *Anaerococcus,* and *Chloroflexi*, were identified as enriched before NACT in the former, while 6, including *Haemophilus*, *Veillonellaceae* (*Veillonella*), and others were enriched after NACT (Figures 1D,E). Thirty-one bacterial taxa, including *Coriobacteriaceae*, *Ruminococcaceae*, *Veillonellaceae,* and *Lachnospiraceae,* were identified in the latter as enriched in R patients. In contrast, 4 taxa, such as *Campylobacterales*, *Epsilonproteobacteria*, etc., were enriched in NR patients (Figures 2D,E). At the phylum level, all were dominated by *Firmicutes*, *Bacteroidetes*, *Proteobacteria*, and *Actinobacteria*. However, there were differences in sequencing results at the family or genus level, which may have occurred due to differences between the controls or between FFPE samples and fresh frozen samples (Greytak et al., 2015).

Notably, we first reported the enrichment of *Chloroflexi* phylum in patients with R before in NACT (Figures 1D,E). The first *Chloroflexi* phylum was described by Pierson et al. (Pierson and Castenholz, 1974) in 1974. However, research evidence linking the *Chloroflexi* phylum to GC in studies is scarce. In a study, Wang et al. (Wang et al., 2020) evaluated the changes in the microbiome during the development of GC. They found that the abundance of *Chloroflexi* phylum from chronic gastritis, through intestinal metaplasia, and intraepithelial neoplasia to GC is gradually decreased. In another study regarding lung cancer, the relative content of *Chloroflexi* phylum was elevated in cancerous tissue samples compared to paracancerous tissue samples (Zhou et al., 2023). Currently, the biochemical roles of the *Chloroflexi* phylum, such as carbon fixation, carbon monoxide oxidation, nitrogen, and sulfur metabolism, have been gradually revealed (Narsing Rao et al., 2022). It has also been demonstrated that the *Chloroflexi* phylum has a nitrate reductase gene (Sanford et al., 2012; McIlroy et al., 2016; Narsing Rao et al., 2022). Nitrite is further reduced to NO by nitrate reductase. Nitrated NO is thought to play an important role in host defense and regulation of gastric mucosal integrity (Petersson et al., 2015). However, nitrate or nitrite is also considered a carcinogen for GC progression (Hernández-Ramírez et al., 2009). Therefore, the link between *Chloroflexi* phylum and GC progression and treatment and whether it can be used as a biomarker for GC patients responding to NACT still needs to be validated in a larger group of subjects. In addition, we found that predominantly bacteria belonging to the family *Veillonellaceae* (genus *Veillonella*) were identified to be enriched after NACT in patients with R (Figures 1D,E). The DELIVER trial results (JACCRO GC-08) suggest that the genus *Veillonella* may be a novel biomarker for effective AGC immunotherapy (Sunakawa et al., 2023). Paradoxically, the genus *Veillonella* appears to be a specific biomarker for detecting and classifying lung cancer (Zhou et al., 2023). Moreover, the genus *Veillonella* can also trigger an inflammatory response through the production of LPS (Delwiche et al., 1985). This is consistent with our functional predictions, noting that LPS biosynthesis appears to be a potential pathway of influence. Consistently, we have also found that the TCA cycle metabolic pathway seems to be suggestive in the comparison of both samples (Supplementary Figures 6, 7), whereas genus *Veillonella* can exert its anti-inflammatory properties by catabolizing lactic acid into propionic and butyric acids via the TCA cycle (Hamilton, 1973; Scheiman et al., 2019). Interestingly, the *Veillonellaceae* family was also identified as enriched in R patients in the analysis of mucosa samples. Similarly, the genus *Veillonella* (Hamilton, 1973) and the phylum *Chloroflexi* (Narsing Rao et al., 2022) were shown to be associated with carbon fixation, consistent with our functional predictions, and we found that carbon fixation pathways in prokaryotes may be potential pathways of influence (Supplementary Figure 7). This evidence seems to suggest that the two samples are somehow congruent. In addition, the genus *Bacillus* has also been found to be enriched in pre-NACT R patients. Some studies have also demonstrated that the genus *Bacillus* is a potentially harmful bacterium of the gastric mucosa (Kadeerhan et al., 2021). Still, contrary studies have confirmed that some species of the genus *Bacillus* also have probiotic value (Acosta-Rodríguez-Bueno et al., 2022). Furthermore, we observed some unclassified species, including the genera *Planococcaceae_incertae_sedis* and *Filomicrobium*.

From the comparison of group mucosa with NR patients, at the family level, we found that several types of bacterial taxa dominated by *Lachnospiraceae*, *Ruminococcaceae*, *Coriobacteriaceae*, etc. were enriched in R patients (Figure 2E). It has been demonstrated that *Lachnospiraceae* and *Ruminococcaceae* species hydrolyze starch and other sugars to produce butyrate and other short-chain fatty acids (SCFAs) (Vacca et al., 2020; Biddle et al., 2023), and SCFAs such as butyrate are thought to be gut-protectors (Bishehsari et al., 2018; Martin-Gallausiaux et al., 2021). In addition, the family *Lachnospiraceae* prevented colorectal carcinogenesis by promoting tumor immunosurveillance (Zhang et al., 2023), and the family *Coriobacteriaceae* is negatively associated with inflammatory bowel diseases (IBD) (Pittayanon et al., 2020), and diabetes mellitus (T2D) (Liu et al., 2018; Zhuang et al., 2021). Consistent with other findings, enriched bacteria identified in R patients at the genus level in this study included *Intestinimonas* (Afouda et al., 2019), *Anaerotruncus* (Lawson et al., 2004), *Roseburia* (Duncan et al., 2002; Vacca et al., 2020), *Coprococcus* (Rogosa, 1969; Vacca et al., 2020), *Megamonas* (Bishehsari et al., 2018), *Alloprevotella* (Bishehsari et al., 2018), and *Acidaminococcus* (Bishehsari et al., 2018; Mager et al., 2020) are producers of SCFAs such as acetic acid and propionic acid. Among them, the genus *Acidaminococcus* and others (Bishehsari et al., 2018; Mager et al., 2020) are involved in the metabolism of amino acids, such as glutamate, consistent with our functional predictions. Moreover, we found that metabolic pathways, including amino acids such as glutamate and alanine, appear to be potential pathways of influence (Supplementary Figure 10). In addition, consistent with our study, the genus *Roseburia* was enriched in R patients in a study with patients with NR as a control, and its optimal abundance variant was shown to be a possible predictor of chemotherapy efficacy in patients with differentiated gastrointestinal tumors (Li et al., 2021). In another study, Mager et al. (2020) demonstrated through mouse experiments that the co-action of the genus *Olsenella* with certain commensal bacteria could further enhance the immunotherapeutic efficacy of CRC by increasing the efficacy of CTLA-4 antibodies. The genus *Gordonibacter* has also been shown to produce urolithins (Kasimsetty et al., 2009) shown to have anti-inflammatory and anticancer properties (Bialonska et al., 2009; Li et al., 2020); the genus *Gemmiger* has been negatively associated with a variety of psychiatric disorders (Ogier et al., 2008). In addition to this, the genus *Leuconostoc* has been generically recognized as safe (GRAS) (Hu et al., 2018); the genus *Sphingobium* is reduced in the gastric microbiota of GC patients (Bessède and Mégraud, 2022), and the genus is capable of degrading xenobiotics compounds, in particular, aromatic hydrocarbons known to have carcinogenic properties (Xie et al., 2018). However, in contrast, the pro-inflammatory bacteria genus *Oscillibacter* (Alexander et al., 2022) and genus *Eggerthella* (Pongen et al., 2023) were shown to be enriched in patients with IBD and worsened colitis in a Rorc-dependent manner in mice; other species were also identified to be enriched in R patients. In contrast, in NR patients, enrichment was dominated by the phylum *Proteobacteria*, which was shown to be tumor-promoting (Marchesi et al., 2016); however, we also observed enrichment of the butyric acid-producing bacterium genus *Butyrivibrio* (Bishehsari et al., 2018). These phenomena are thought-provoking, and although SCFAs, such as butyrate, have been shown to have a protective effect on the gastrointestinal tract (Bishehsari et al., 2018; Zhang et al., 2023), they also exert a pro-tumorigenic role (Marchesi et al., 2016). Notably, bacterial diversity has been an obstacle for researchers to discover potential cancer development or treatment biomarkers. However, this does not mean that the possibility of utilizing microorganisms to participate in disease progression and treatment can be ruled out, which requires more in-depth basic research and larger preclinical or clinical trials to conclude.

Limitations of this study should be recognized. On the one hand, this study was a retrospective study with a small sample size, which may have contributed to our inability to explain some of the phenomena. On the other hand, the use of 16 s sequencing technology alone does not allow for good annotation and requires the combined use of technologies such as metagenomics, metabolomics, etc., and the need to move away from simple observation to the determination of correlation to determine whether it is purely correlational, or causation.

## Conclusion

The data in the study confirm that NACT response in GC patients is associated with GM. However, further experiments are needed to prove the causal or consequential relationship. In addition, there may be some consistency between FFPE samples and fresh frozen samples, and the study found that the *Veillonellaceae* family was enriched in both samples of R patients. More importantly, the study reported for the first time the enrichment of *Chloroflexi* phylum before NACT in R patients, which was not available in previous studies. However, whether these two have the biomarker potential remains to be proved by further experiments.

In summary, the study confirms that GM may be involved in NACT in GC patients and also provides a theoretical basis for the next step of basic research and individualized treatment of GC.

## Data availability statement

The datasets presented in this study can be found in online repositories. The names of the repository/repositories and accession number(s) can be found at: https://osf.io/r634v/, PRJNA1068298.

## Ethics statement

The studies involving humans were approved by Ethics Committee of the Affiliated Hospital of Qingdao University. The studies were conducted in accordance with the local legislation and institutional requirements. The participants provided their written informed consent to participate in this study.

## Author contributions

PZ: Conceptualization, Data curation, Formal analysis, Funding acquisition, Investigation, Methodology, Project administration, Resources, Software, Supervision, Validation, Visualization, Writing – original draft, Writing – review & editing. JX: Conceptualization, Methodology, Project administration, Resources, Supervision, Validation, Writing – review & editing. YZ: Conceptualization, Funding acquisition, Methodology, Project administration, Resources, Supervision, Writing – review & editing.
