# Supplementary material for: The relationship between gastric microbiome features and responses to neoadjuvant chemotherapy in gastric cancer
Source: Front Microbiol. 2024 Apr 17;15:1357261. doi: 10.3389/fmicb.2024.1357261 (PMC11061454; doi:10.3389/fmicb.2024.1357261)
Supplement: Supplementary file 1 [file Table_1.PDF]

Supplementary table 1. Comparison of bacterial taxa in gastric microbiome before and after NACT by STAMP.

Taxon	Relative Abundance before NACT (%)	Relative Abundance after NACT (%)	*P*-values	Taxonomy level
Bacteroidota	0.647312	1.076729	0.032	Phylum
Alphaproteobacteria	10.740687	12.182471	0.038	Class
Flavobacteriia	0.183402	0.500098	< 0.05	Class
Rhodobacterales	1.176454	1.959503	0.035	Order
Paracoccaceae	0.978407	1.749271	0.038	Family
Paracoccus	0.901847	1.656614	0.046	Genus
