# Supplementary figures and images for: The relationship between gastric microbiome features and responses to neoadjuvant chemotherapy in gastric cancer
Source: Front Microbiol. 2024 Apr 17;15:1357261. doi: 10.3389/fmicb.2024.1357261 (PMC11061454; doi:10.3389/fmicb.2024.1357261)

**A**

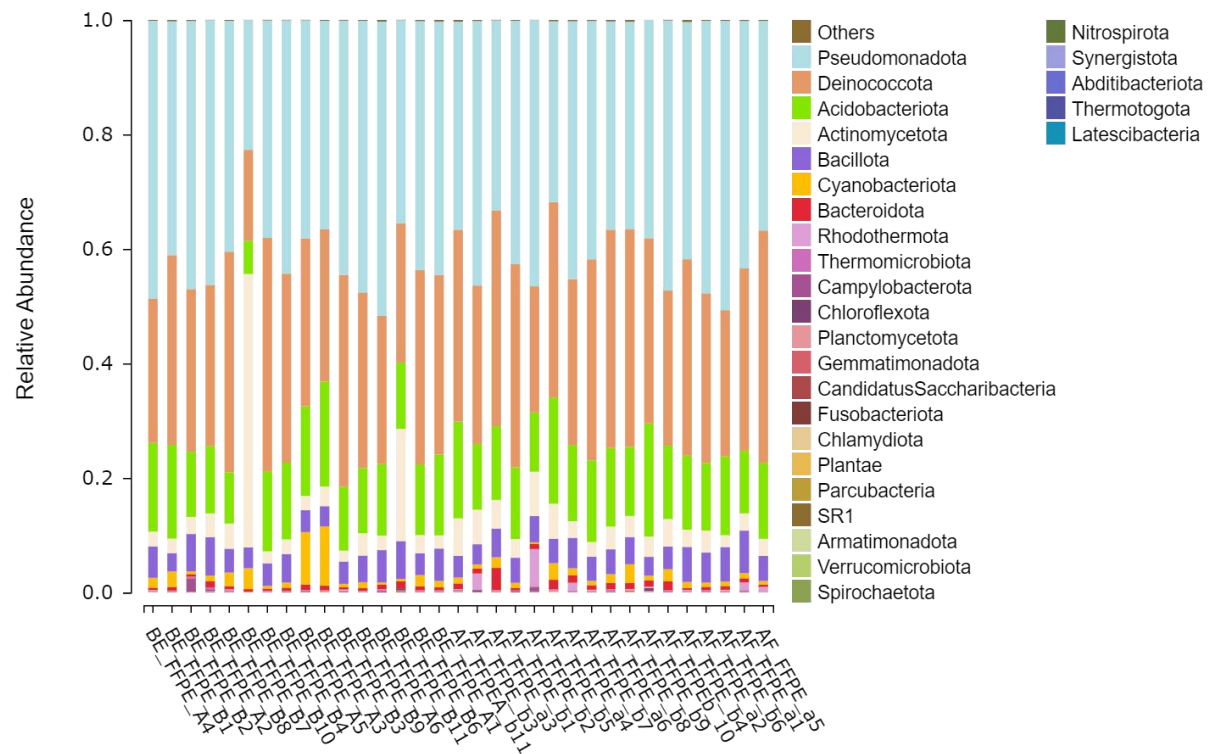

**B**

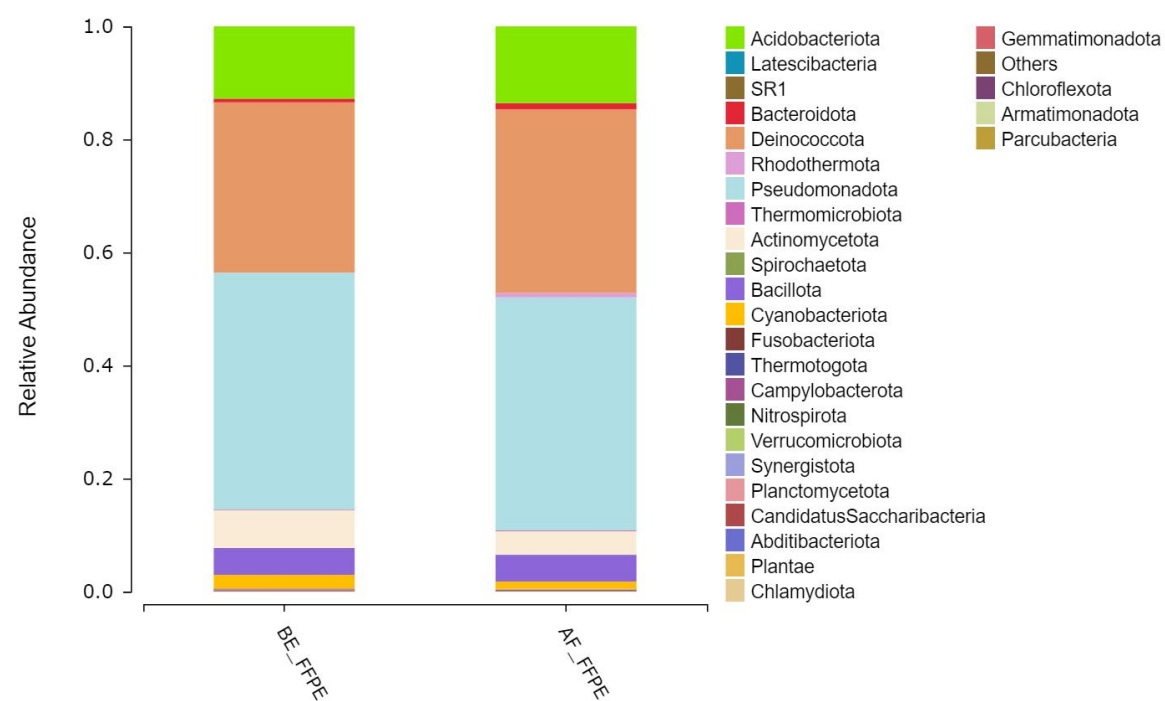

Supplement: Supplementary file 2 [file Image_1.PDF]

**A**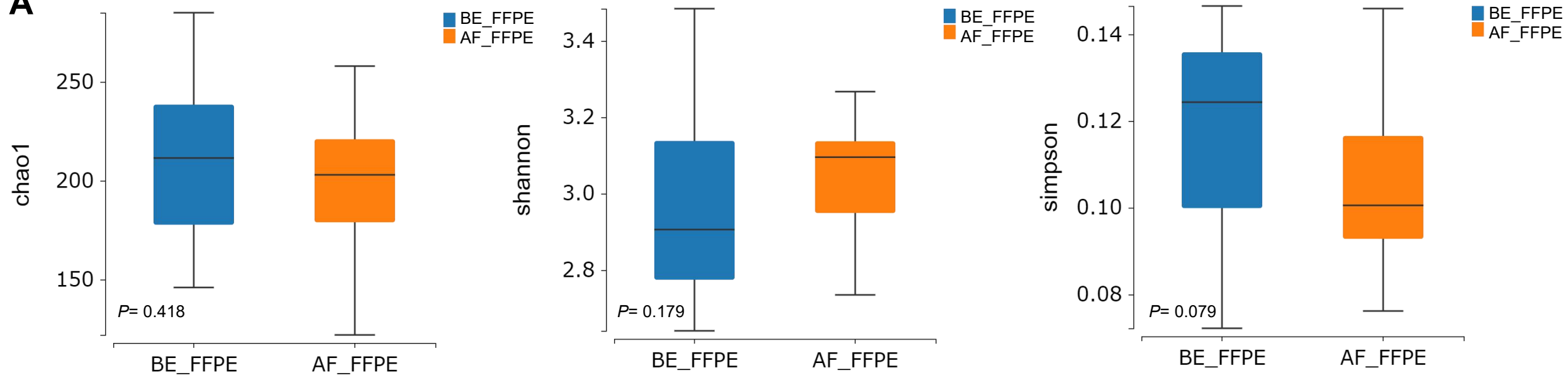**B**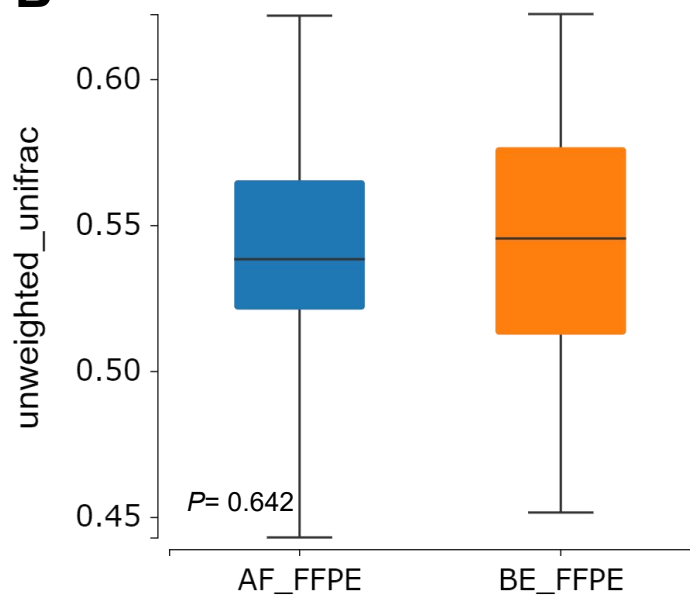**C**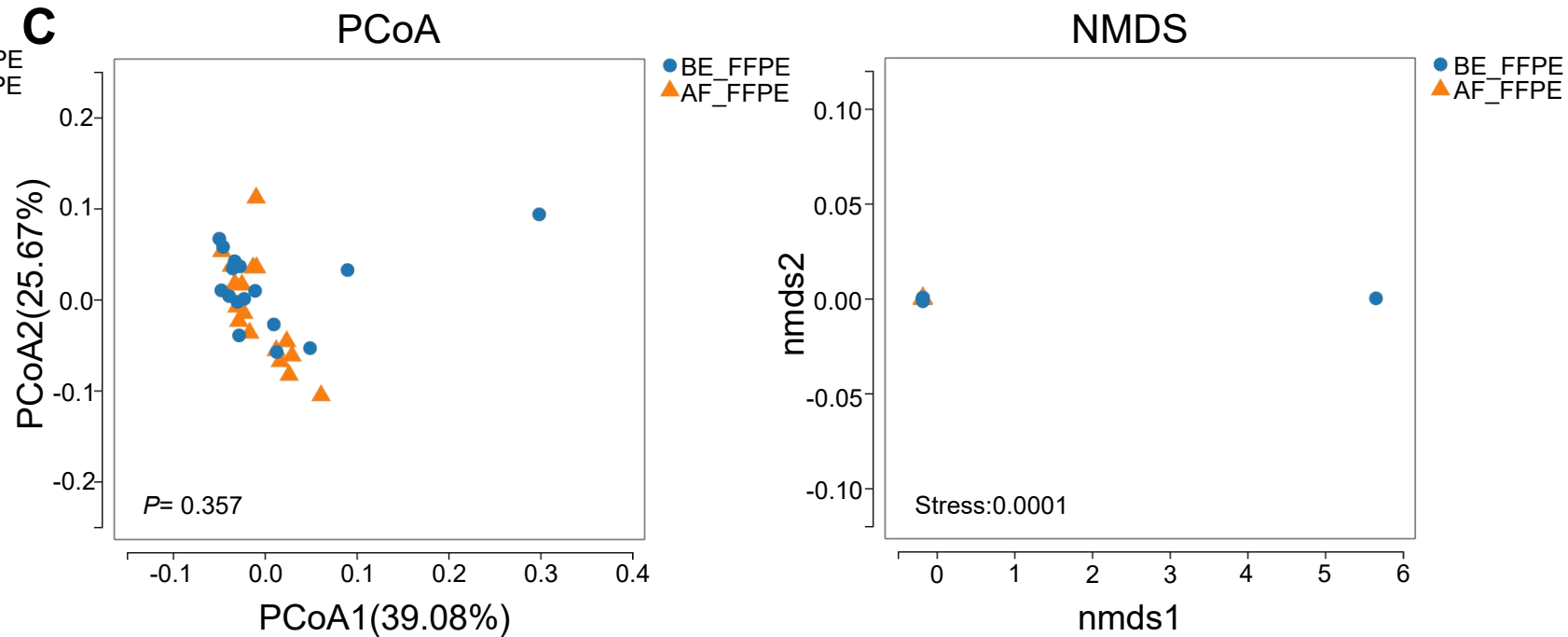

Supplement: Supplementary file 3 [file Image_2.PDF]

A

## Cladogram

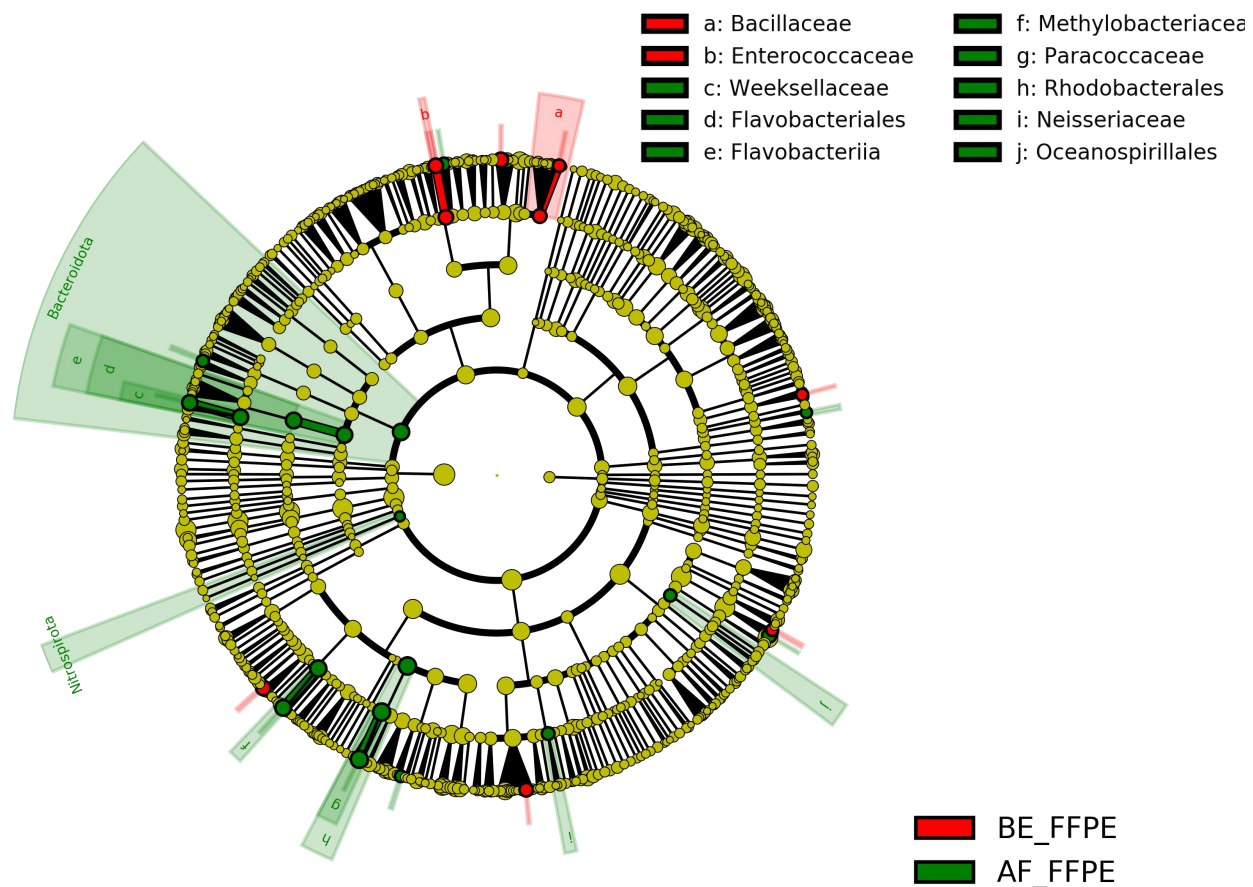

B

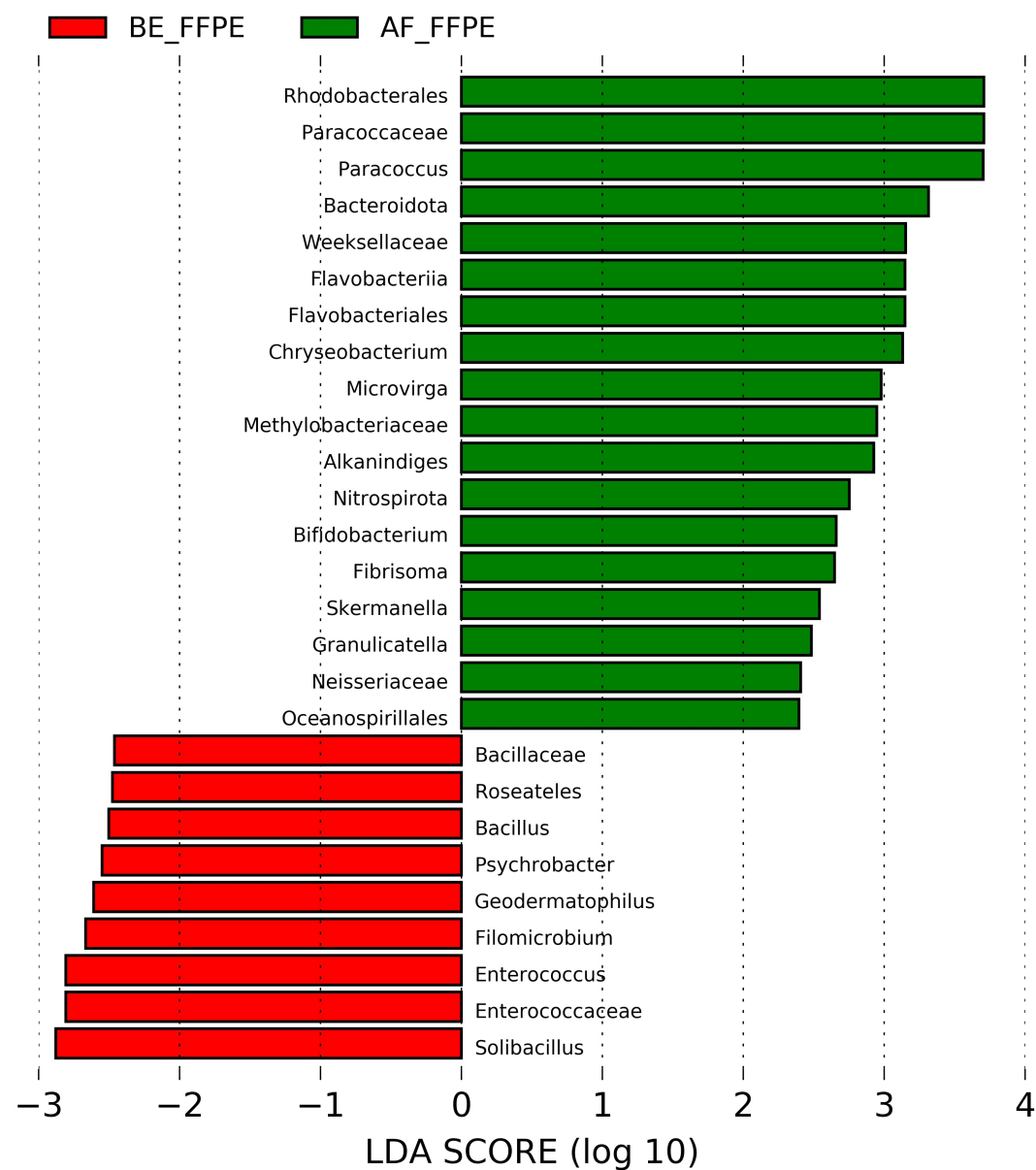

Supplement: Supplementary file 4 [file Image_3.PDF]

**A**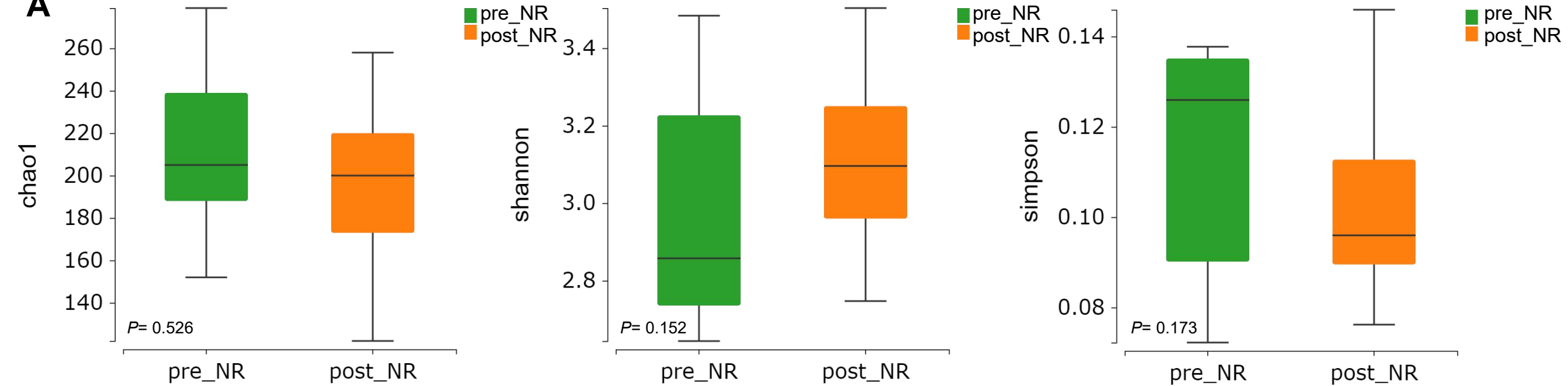**B**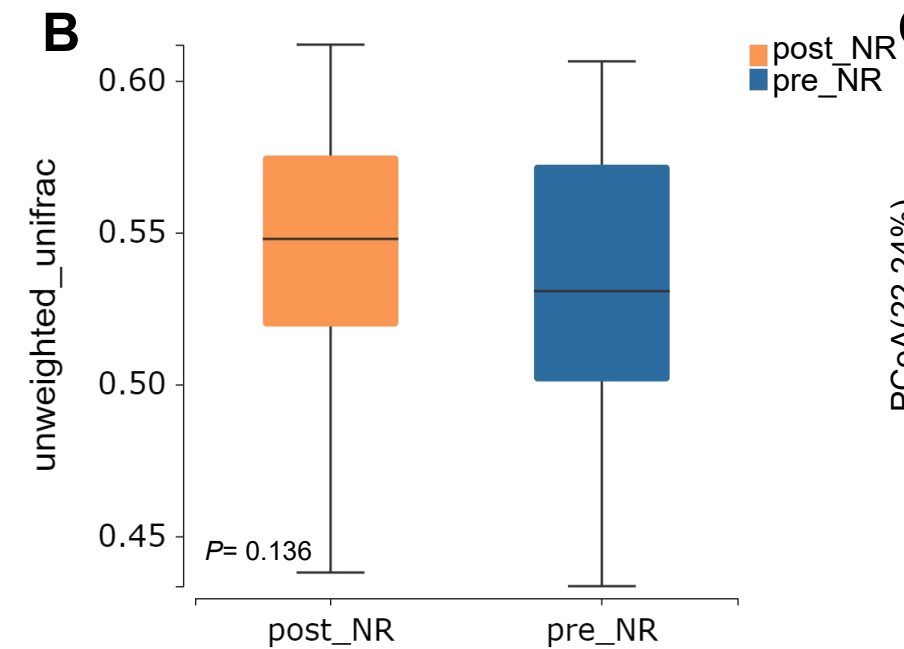**C**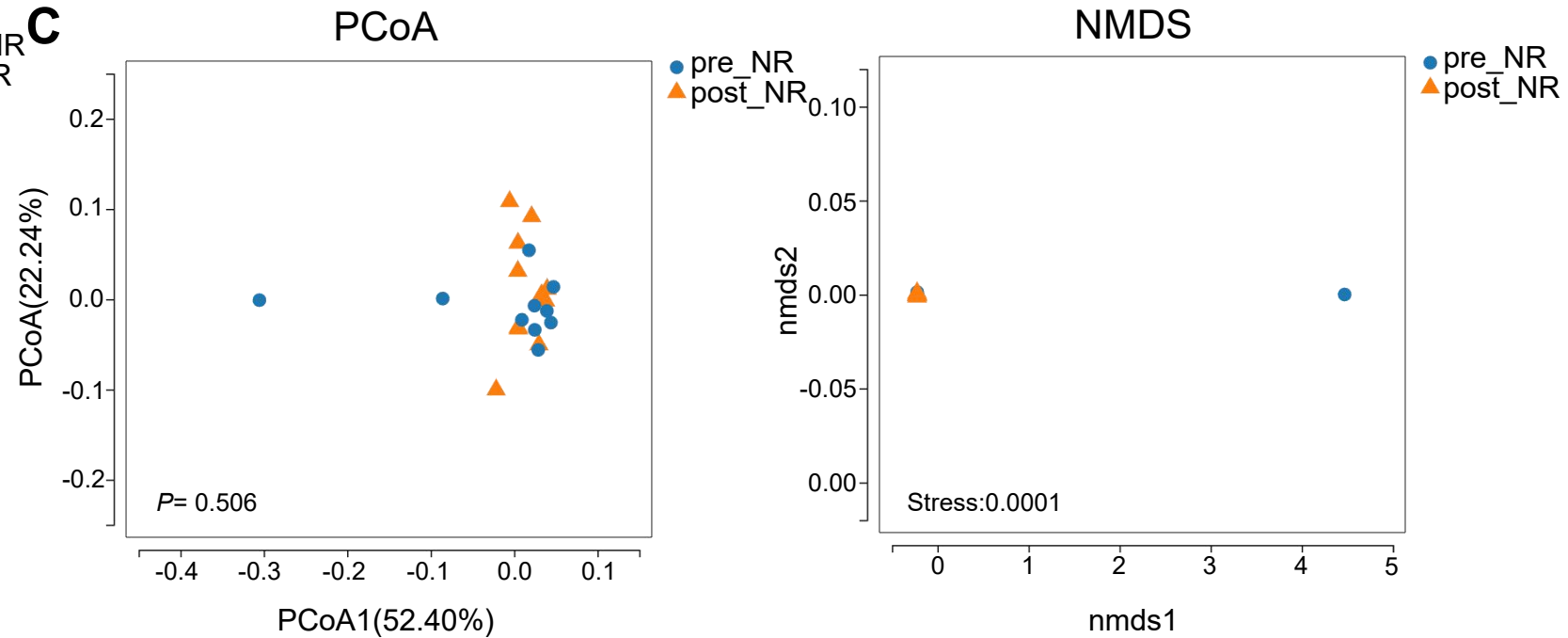

Supplement: Supplementary file 5 [file Image_4.PDF]

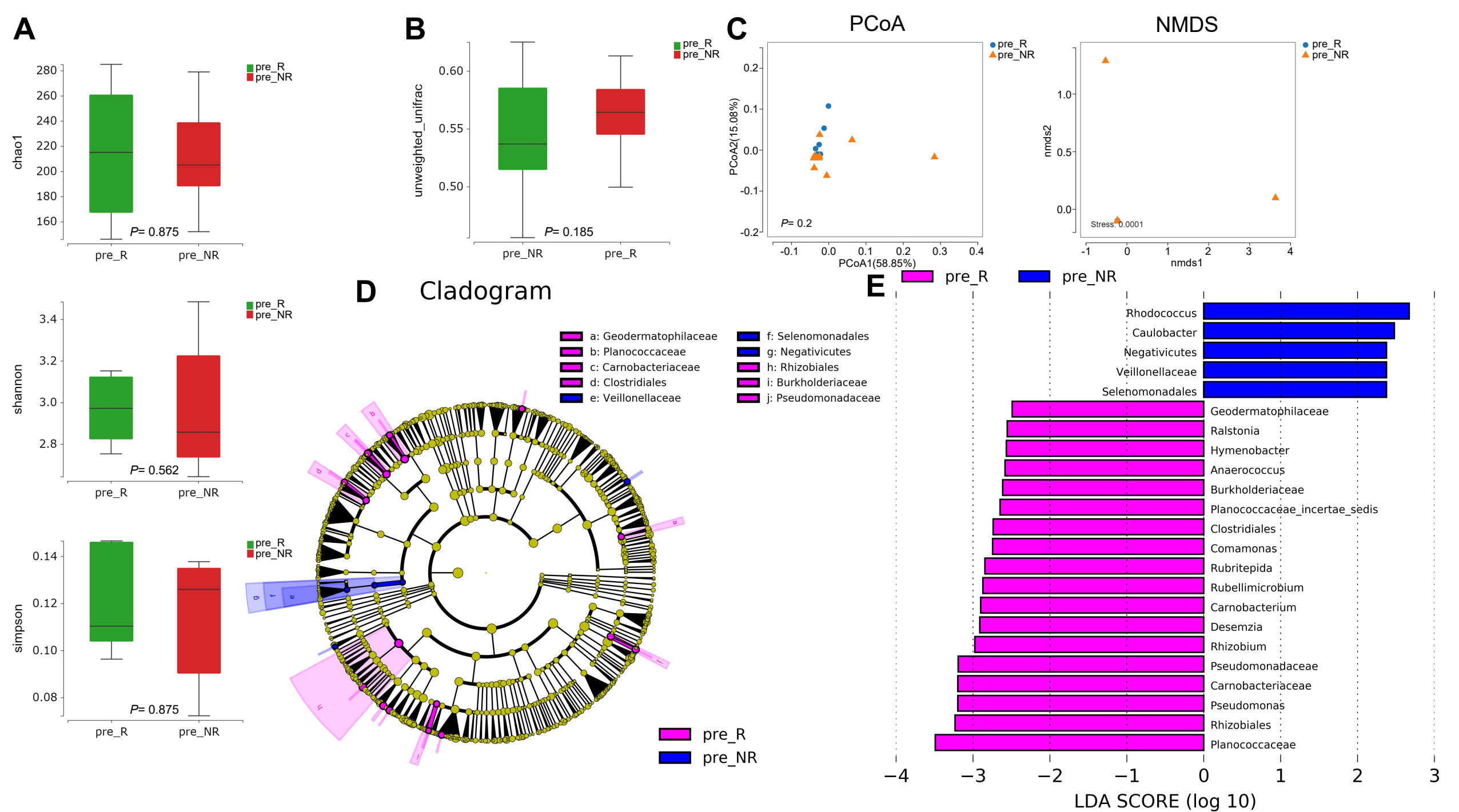

Supplement: Supplementary file 6 [file Image_5.PDF]

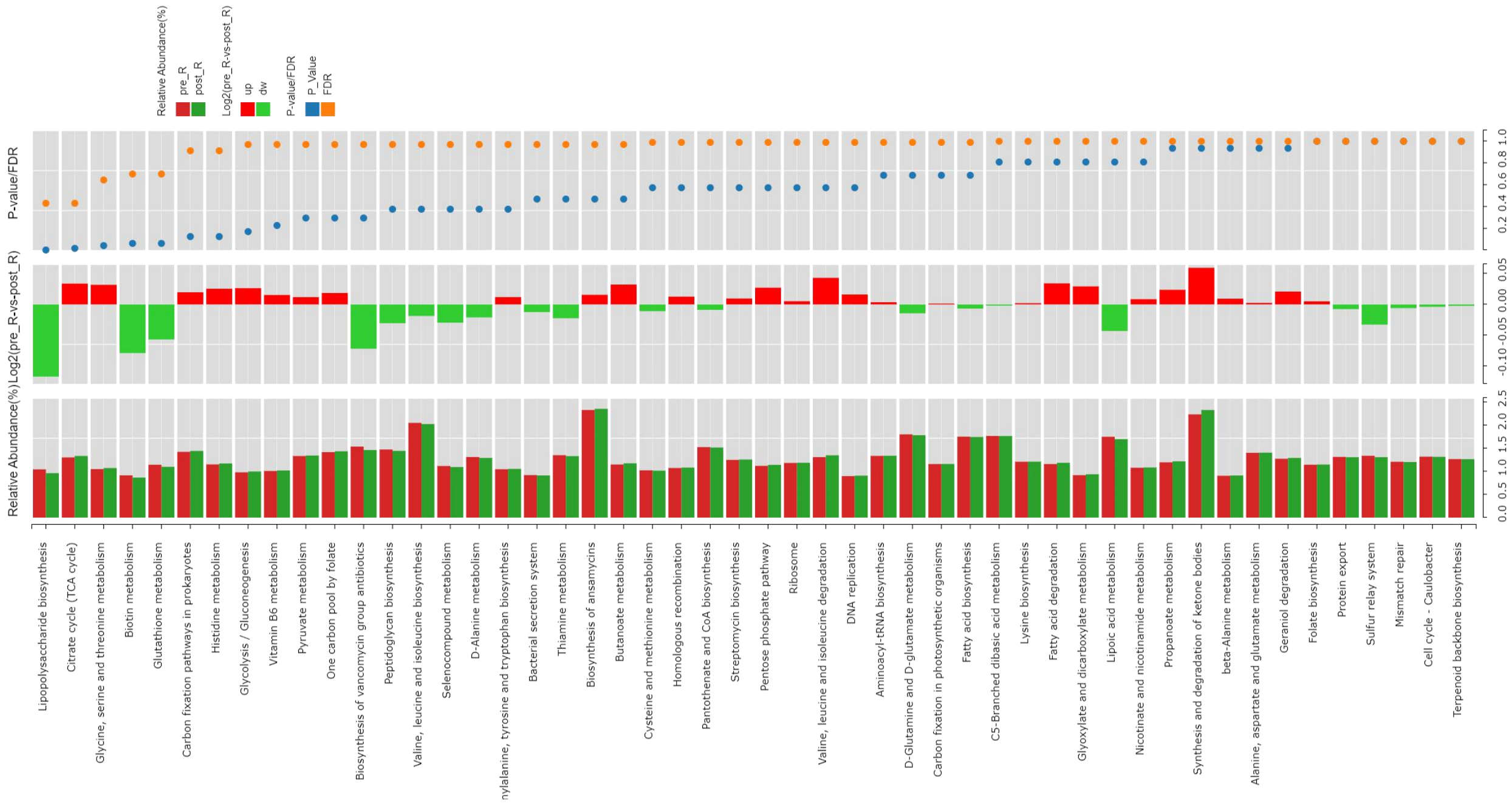

Supplement: Supplementary file 7 [file Image_6.PDF]

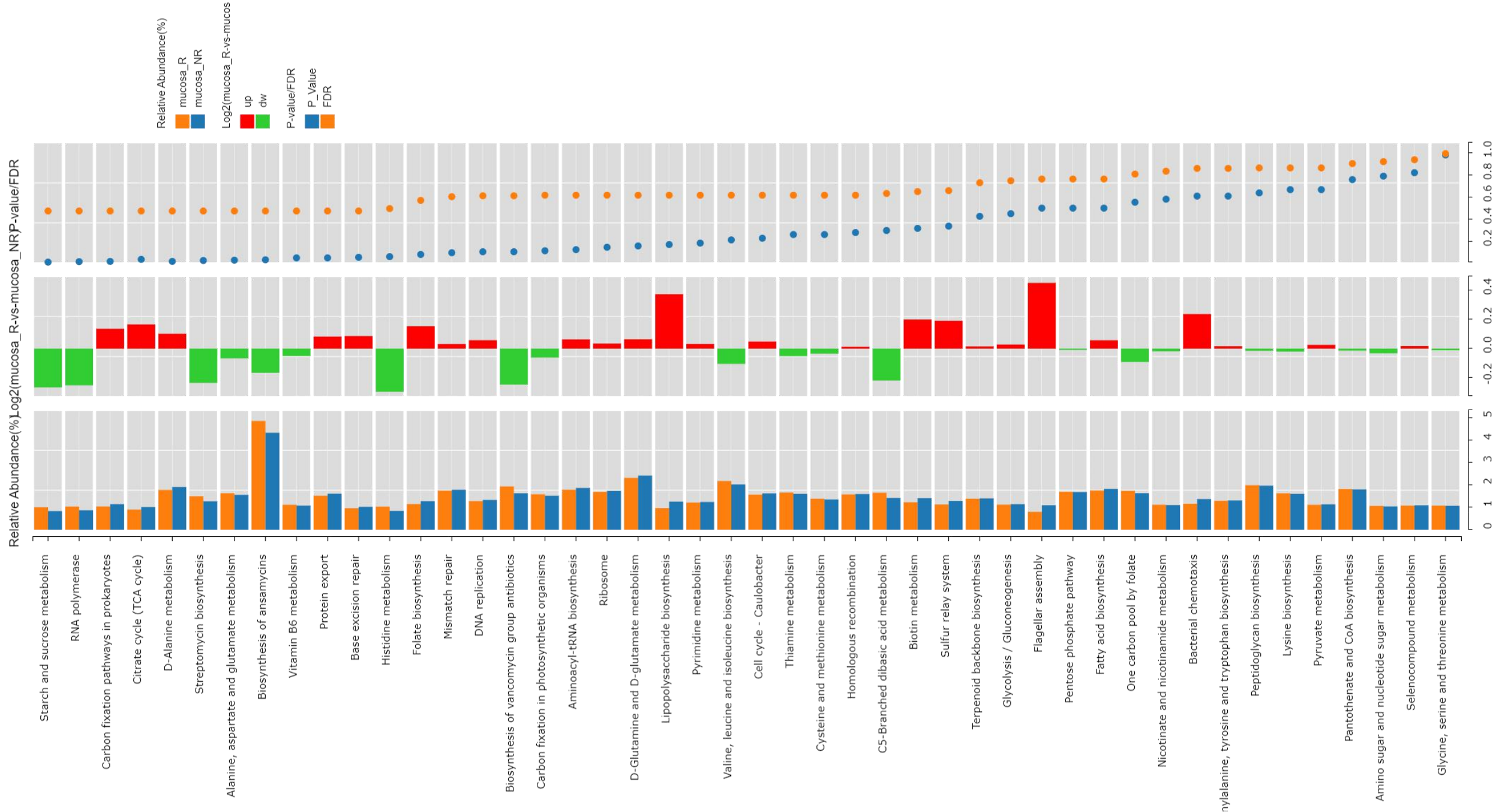

Supplement: Supplementary file 8 [file Image_7.PDF]
